# Class Prediction and Feature Selection with Linear Optimization for Metagenomic Count Data

**DOI:** 10.1371/journal.pone.0053253

**Published:** 2013-03-26

**Authors:** Zhenqiu Liu, Dechang Chen, Li Sheng, Amy Y. Liu

**Affiliations:** 1 University of Maryland Greenebaum Cancer Center, Baltimore, Maryland, United States of America; 2 Department of Preventive Medicine and Biometrics, Uniformed Services University of the Health Sciences, Bethesda, Maryland, United States of America; 3 Department of Mathematics, Drexel University, Philadelphia, Pennsylvania, United States of America; 4 Department of Applied Math, Brown University, Providence, Rhode Island, United States of America; The University of Queensland, Australia

## Abstract

The amount of metagenomic data is growing rapidly while the computational methods for metagenome analysis are still in their infancy. It is important to develop novel statistical learning tools for the prediction of associations between bacterial communities and disease phenotypes and for the detection of differentially abundant features. In this study, we presented a novel statistical learning method for simultaneous association prediction and feature selection with metagenomic samples from two or multiple treatment populations on the basis of count data. We developed a linear programming based support vector machine with 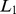 and joint 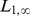 penalties for binary and multiclass classifications with metagenomic count data (metalinprog). We evaluated the performance of our method on several real and simulation datasets. The proposed method can simultaneously identify features and predict classes with the metagenomic count data.

## Introduction

The majority of microbes reside in the gut, have a profound influence on human physiology and nutrition, and are crucial for human life. Metagenomics, the culture-independent isolation and characterization of DNA from uncultured microorganisms, has facilitated the analysis of the functional biodiversity harbored in the large reservoir of uncultured bacteria and archaea. The goals of microbiome research are to delineate the host-microbiota interactions, associate differences in microbial communities with differences in metabolic function and disease, and understand how changes in the microbiota may affect human health. Recent advances in genome sequencing technologies have made obtaining a complete metagenomic sequencing more tractable [1]. Having on hand such a large number of microbial genomes has changed the nature of microbiology and of microbial evolution studies. By providing the ability to examine the relationship of genome structure and function across many different species, these data have also opened up the fields of comparative genomics and of systems biology [2], [3]. A main promise of metagenomics is that it will accelerate drug discovery and biotechnology by providing new genes with novel functions [2], [4].

In metagenomics, one aim is to understand the composition and operation of complex microbial assemblages in both human and environmental samples through sequencing and analysis of their DNA. There have been great efforts in determining the taxonomical and functional contents of a sample in the last several years. One way is to use a homology-based approach, which is based on comparing the sequencing reads against a reference database such as the NCBI-NR database of nonredundant protein sequences [5], usually employing a variant of the program BLAST [6]. The result of this extensive computation is a set of high-scoring pairs or matches that represent possible homologies between genes in the data set and genes in the reference database. This must then be analyzed so as to obtain a taxonomic profile and/or functional profile for the input data. Several tools employ a homology-based approach, including MEGAN [3], [7] MG-RAST [8], IMG/M [9], CAMERA [10], and CARMA3 [11]. An alternative to a homology-based approach is to employ a machine-learning method that uses simple signatures of the reads, as implemented in TETRA [12], PhyloPythia [13], and PhyloPythiaS [14]. More recent tools include Phymm and PhymmBL [15], NBC [16], PCAHIER [17], and INDUS [18]. The NB-based classification approach which hybridizes both homology and composition was also proposed [19]. There are a number of tools that focus primarily on the analysis and comparison of 16S and 18S data, such as MOTHUR [20], MLtreemap [21], UniFrac [22], QIIME [23], and CloVR [24]. Those softwares provide different approaches for taxonomic classification of metagenomic sequence data. The ultimate goal, however, is to identify specific microbia and microbial communities that are associated with human diseases. Comparing metagenomes from two or more populations with different disease statuses is necessary for understanding how genomic differences affect, and are affected by, the abiotic environment, but study of the link between characteristics of microbiome and disease status is in its infancy. Thus, there are not many methods for studying the associations and interactions between metagenomic data and clinical outcomes.

Statistical test based approaches such as MetaStats [25] were designed to compare one microbial feature at a time and can not be used to identify multiple features simultaneously. Moreover, we do not know the prediction power of those identified features, which is very important in clinical metagenomic research. Investigators want to know how strong the association is between microbial features and clinical phenotypes. Supervised learning methods such as support vector machines (SVM) have been extensively studied with gene expression data [26] and they have been applied to classify psbA fragments based on genomic composition in the marine environment [27]. Linear programming (LP) is a branch of mathematical programming with linear constraints and an objective function. It has found applications in many research fields including microarray analysis [28]–[30]. However, those approaches were mainly formulated as binary classification problems without the ability to select features and predict classes simultaneously. In this paper, we propose a novel supervised learning method using LP based support vector machine (SVM) with joint 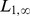 penalty for simultaneous feature selection and binary/multiclass prediction. Our proposed method identifies common microbial features for multiclass predictions, which overcomes the drawback that different classifiers choose different features when applying the one-against-rest rule for multiclass prediction. We evaluate the performance of our tool (metalinprog) through simulation, publicly available, and our own metagenomic data sets. The proposed methods are robust across datasets and efficient for microbial feature identification and phenotype prediction. The software metalinprog is implemented in MATLAB and is available at http://biostatistics.csmc.edu/metalinprog/.

## Methods

To understand the association between the metagenomic contents and clinical phenotypes such as cancer, it is crucial to develop new supervised learning tools. We assume there are two or multiple populations with different clinical phenotypes (e.g. cancer and healthy, or different treatments) and each has multiple samples. For each sample we have multiple metagenomic count features including the number of 16S rRNA clones assigned to a specific taxon, or number of shotgun reads mapped to a specific biological pathway or subsystem as shown in the follows:
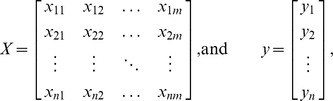
where X is the metegenomic count matrix with n samples and m features, 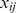 denotes the total number of reads of feature 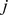 in sample 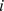, and y is the clinical phenotypes with 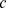 categories. 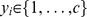. Our goals are to identify features whose abundance in different populations is different, and estimate the power of those identified features in predicting clinical phenotypes.

There are two sources of bias in the metagenomic count data: (1) different levels of reads (sampling) across multiple samples, and (2) the variance of 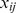 depends on its particular value. Validity of many statistical procedures relies upon the assumptions of normal distribution and homogeneity of variances. However, the metagenomic count and related percentage data have variances that are a function of the mean and are not normally distributed but instead are described by Poisson, binomial, negative binomial, or other discrete distributions. The variance heterogeneity and non-normality of the metagenomic count data can seriously increase either Type I or II error and make the statistical inferences invalid. Therefore, The following data preprocessing and variance-stabilizing transformation steps are required before we build predictive models for metagenomic data classification:

1. Converting the raw abundance measure of each sample to the relative abundance to adjust for the sampling depth (read count) differences across samples. Mathematically, we normalize the metagenomic count matrix 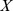 into a relative abundance matrix 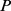 with
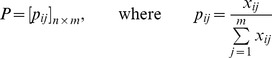



2. We then employ either the square root transformation or the arcsine transformation to the relative abundance matrix P:

•Square root transformation:
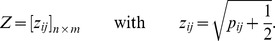



•Arcsine transformation:
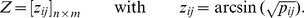



Before we do any transformations, we will compute the mean and variance for each sample with matrix 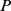 or 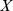, and then test the assumption of homogeneity of variances with Bartlett's test [31]. Either the square root or arcsine transformation will be used. Practically, if the percentage data have homogeneous variances, no transformation is needed. For data with variance heterogeneity, if the data lie in the range of 0–0.3 or 0.7–1 but not both, the square root transformation should be used. Otherwise, the arcsine transformation should be used. In most cases, we find both transformations increase predictive power and have similar performance [32]. In this paper, we therefore utilize the arcsine transformation with proportion data for all of our experiments.

### 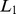 and 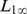 Penalized SVM Methods

#### 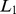 Penalized SVM Method with Linear Programming

When there are two classes (number of categories 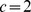), a general binary classification problem may be simply described as follows. Given n samples, with normalized features, 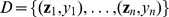 , where 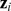 is a multidimensional feature vector with dimension 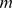 and class label 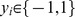, find a classifier 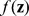 such that for any normalized feature vector 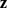 with class label y, 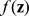 predicts class y correctly. Consider the case of learning a single sparse classifier on the normalized feature space of the form:
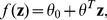
(1)where 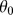 is the intercept and 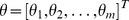 are the coefficients (parameters). A sparse model will have a small number of features with nonzero coefficients. A natural choice for the sparse models is to find optimal parameters 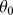 and 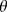 that minimize the following 
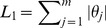
 penalized loss function:
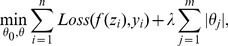
(2)where the left term measures the error that the classifier incurs on training examples measured in terms of loss function, and the right term is the 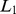 penalty which encourage sparsity, where the larger the 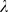, the more sparse the model. Naturally, we penalize parameters associated with each normalized feature without penalizing the intercept term 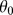. The loss function for soft-margin SVM is defined as



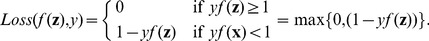
(3)The 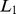 SVM, therefore, identifies the phenotype associated features and evaluates the model predictions by optimizing
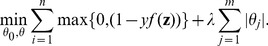
(4)


Equation (4) can be reformulated as following linear program:
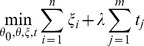


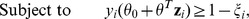


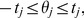
(5)

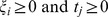


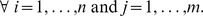



#### Multiclass SVM with Joint 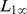 Penalty

We adopt the common technique of representing the class labels using the `one-against-rest' role for general multiclass (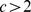) problems. We encode each 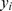 into a vector 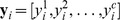 such that 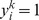 if 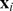 belongs to class 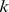 (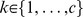), and 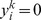 otherwise. After encoding, a multiclass problem becomes 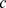 binary class problems. We have the parameter of 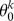 and 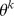 for the binary model 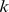. There are a total of 
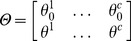
 parameters to be estimated, where 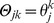 corresponds to the 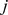-th coefficient of the 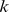-th problem (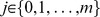). In this way, the 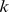-th problem is defined as 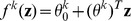. Our goal is to identify the most discriminative microbes for the clinical phenotypes. Clearly the number of non-zero rows of 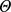 corresponds to the total number of microbes selected by any of the 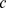 classifiers. This suggests learning the sparse optimization problem jointly across rows of 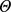, which overcomes the vital drawback that different binary classifiers select different microbe features if we optimize the 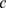 binary classifiers separately. The 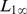 has been applied in multi-task learning for joint feature selection [33]–[35]. It is defined as
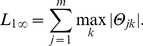
(6)


The 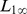 promotes joint sparsity by combining an 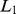 norm and 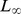 norm on the coefficient matrix 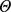. The 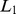 norm operates on a vector formulated by the maximal absolute values of the coefficient of each microbial feature across problems, encouraging most of these values to be 0. On the other hand, the 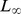 norm on each row promotes non-sparsity among the coefficients of a feature. As long as the maximal absolute value is not affected, no penalty is incurred for increasing the values of a row's coefficient. As a result only a small subset of discriminative microbes will be selected in our model and the identified microbes will contribute to joint multiclass prediction problems. Based on 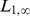 and similar to equation (4), we define the following joint learning problem for multiclass SVM:
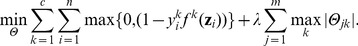
(7)


Equation (7) is equivalent to the following linear optimization problem:
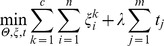


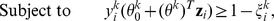


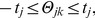
(8)

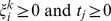


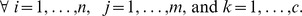



The second constraint in Equation (8) bounds the coefficients for the 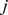-th feature across c problems to lie in the range of 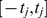. Usually it is better to transform the row score 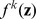 to probability with
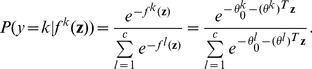
(9)


The final class prediction for each sample is determined by 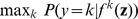. Because the normalization condition 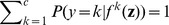, the parameters for one of the classes need not to be estimated. Without loss of generality, we thus set 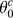 and 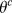 to zero. For the remainder of the paper, we estimate 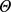 as a 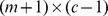 matrix.

#### Algorithms and Choice of Parameter 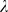


The huge advantage of our linear programming based SVM approach is that it can find a globally optimal solution with an off-the-shelf package. Efficient algorithms for linear programming are available in literature [36], [37]. The non-commercial linear programming code of choice appears to be lp_solve, written in ANSI C by Michel Berkelaar, who claims to have solved problems with as large as 30,000 variables and 50,000 constraints (http://lpsolve.sourceforge.net/5.5/). Matlab also has a linprog function in its optimization toolbox. Efficient large-scale interior point algorithm is implemented in both functions. The regular parameter 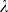 controls the sparsity of the model. The larger the 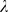, the fewer the microbial features to be selected. If 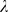 is too small, there will be overfitting and little sparsity. If 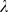 is too large, the produced classifier will be very sparse but have poor predictability. The optimal 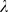 is chosen with the smallest test error through 10-fold cross validation.

## Results

### Simulation Data

**Figure 1:**
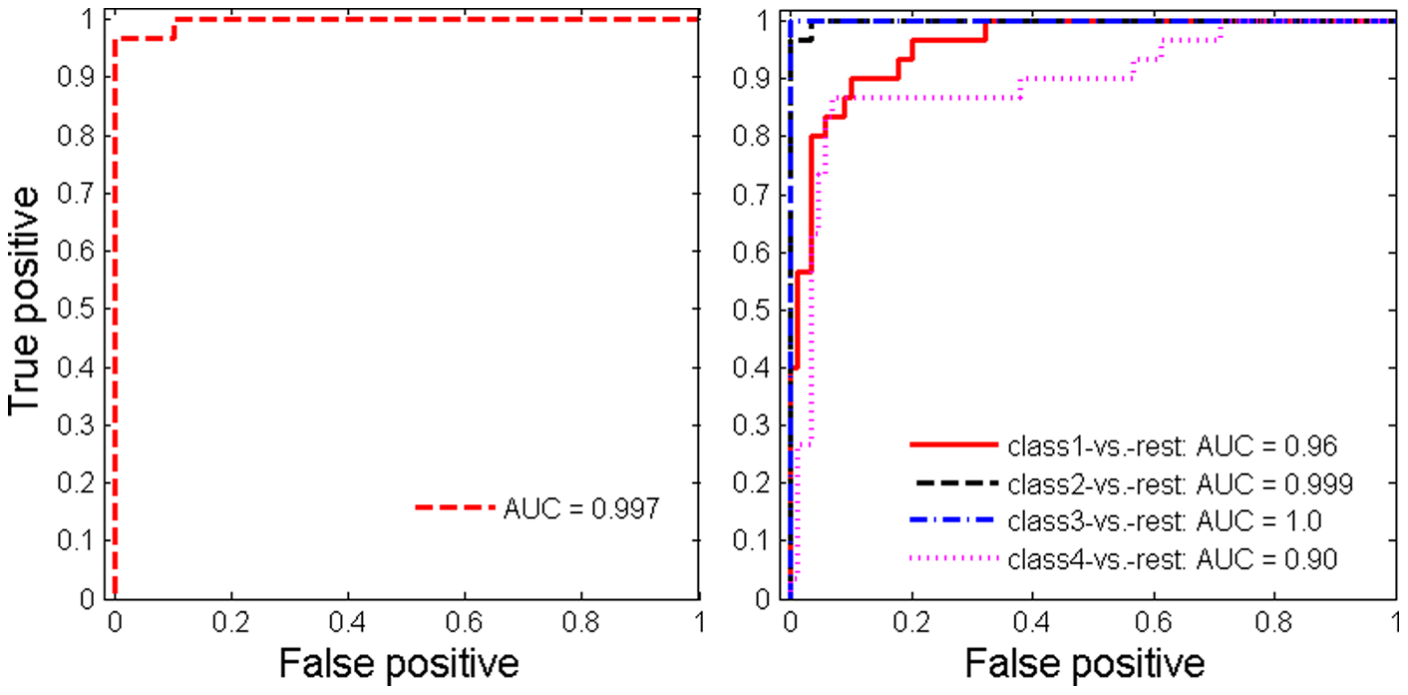
Test ROC curves and AUCs for simulation data: Left: 2-Classes; Right: 4-Classes.

We first evaluate our proposed methods using simulated metagenomic count data with 2 and 3 different classes, respectively. The datasets with the sample size of 50 for each class are generated using Poisson distributions with different means (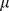
s). The means (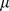
s) for Poisson distributions are simulated from the Gamma distribution with a mean (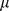) of 100 and variance (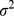) of 1000. We simulated 1000 features for each sample from NB distributions, which contained the first 5 relevant features having different distributions with distinguished 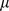
s. We used two-fold cross validation to evaluate the method. First, we normalized the data with proportion and arcsin transformations, and then divided the data into training and test equal subsets. The training subset was used for model construction, while the test subset was used to evaluate performance. The model parameters 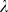 are determined from only the training data with leave-one-out cross-validation. To prevent bias arising from a specific partition, we simulated the datasets of each sample size 100 times. The optimal 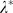
s are 5 and 7 for the binary and 4-class classifications respectively. The frequencies of correctly identified features for 2-class and 4-class predictions are reported in Table 1 and the ROC curves for the test data are given in Figure 1.

**Table 1:** Frequencies of Correctly Identified features with Different numbers of classes.

Features	2-Classes	4-Classes
1	99	96
2	100	97
3	97	100
4	100	100
5	100	99
Av. # of Features	4.9	4.86

Both Figure 1 and Table 1 show that metlinprog performs well in both binary and multiclass classification. With a sample size of 50 for each class, the 5 class associated features are identified with over 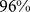 accuracy and the average number of features selected are 4.9 and 4.87 respectively, which are very close the the number of true features (5). The average test AUCs are 0.997 and 0.97 for the binary and 4-class classifications, respectively. The proposed approach performs better than the multinomial logistic regression (mlogit) R package (http://cran.r-project.org/web/packages/mlogit), which has the average predictive AUCs of 0.97 and 0.94 for the binary and 4-class classifications, respectively.

### Hand Surface Bacteria Data

Bacteria thrive on and within the human body. One of the largest human-associated microbial habitats is the skin surface, which harbors large numbers of bacteria that can have important effects on health [38]. This data was collected for characterizing bacterial diversity on hands and assessing its variability within and between individuals. The palmar surfaces of the dominant and nondominant hands were examined from approximately 93 undergraduate students in two different studies. Sequences were processed and analyzed following the standard processing pipeline [38]. Operational taxonomic unit (OTU) count data were generated using Mothur package ([20], PMID: 19801464) at a sequence similarity threshold of 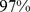. The total group method in Mothur was used to find the normalized abundance. There are total 175 metagenomic data samples without missing values. We intend to predict the gender of the samples and identify gender associated OTUs simultaneously. We first normalized the data with proportion and arcsine transformation, and then evaluated the model performance with two-fold cross validation. To prevent bias arising from a specific partition, we divided the data into roughly-equal two parts (one as the training and the other as the test data) 100 times through permutation. The free parameter 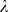 is determined through cross-validation with the training data only. The optimal 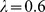. The relevance count is calculated by the number of times an OTU is selected in 100 permutations. The selected OTUs are reported in Table 2. The numbers in the parentheses are the relevance counts for that OTU being selected.

**Table 2:** Identified OTUs for hand surface bacteria data.

Firmicutes;“Bacilli”; “Lactobacillales”;Lactobacillaceae;Lactobacillus (100)
Proteobacteria;Gammaproteobacteria;Pseudomonadaceae;Pseudomonas(83)
Firmicutes; “Bacilli”; “Lactobacillales”;Streptococcaceae;Streptococcus (100)
Proteobacteria;Betaproteobacteria;Neisseriales;Neisseriaceae;Neisseria (78)
Firmicutes; “Bacilli”;Bacillales; “Listeriaceae”;Brochothrix (76)
Firmicutes; “Bacilli”; “Lactobacillales”;Streptococcaceae;Lactococcus (100)
Firmicutes; “Bacilli”;Bacillales; “Staphylococcaceae”;Staphylococcus (100)
Proteobacteria;Betaproteobacteria;Burkholderiales;Comamonadaceae;Acidovorax (92)
Proteobacteria;Betaproteobacteria;Burkholderiales;Incertae sedis 5 (100)

We evaluate the performance of MetClass through comparing with logistic regression (mlogit). The proposed approach achieves the test AUC of 0.81 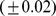 and predictive error of 0.22 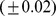 with only 9 OTUs, which is better than the best performance with logistic regression ( test AUC 0.73 and predictive error 0. 31) with all OTUs. Among the 9 identified OTUs, 5 OTUs are from the Firmicutes family and 4 are from Proteobacteria. The relative abundances of those 9 OTUs are different between men and women, which indicate men and women harbor significantly different bacterial communities on their hand surfaces. Both Lactobacillaceae and Pseudomonadaceae were also reported statistically significant in the original study. There are several possible factors driving those differences in bacterial diversity. Differences in skin PH, sweat or sebum production, frequency of moisturizer or cosmetics application, skin thickness, and hormone production can all contribute to distinct hand bacterial communities in men and women.

### Keyboard Dataset

**Figure 2:**
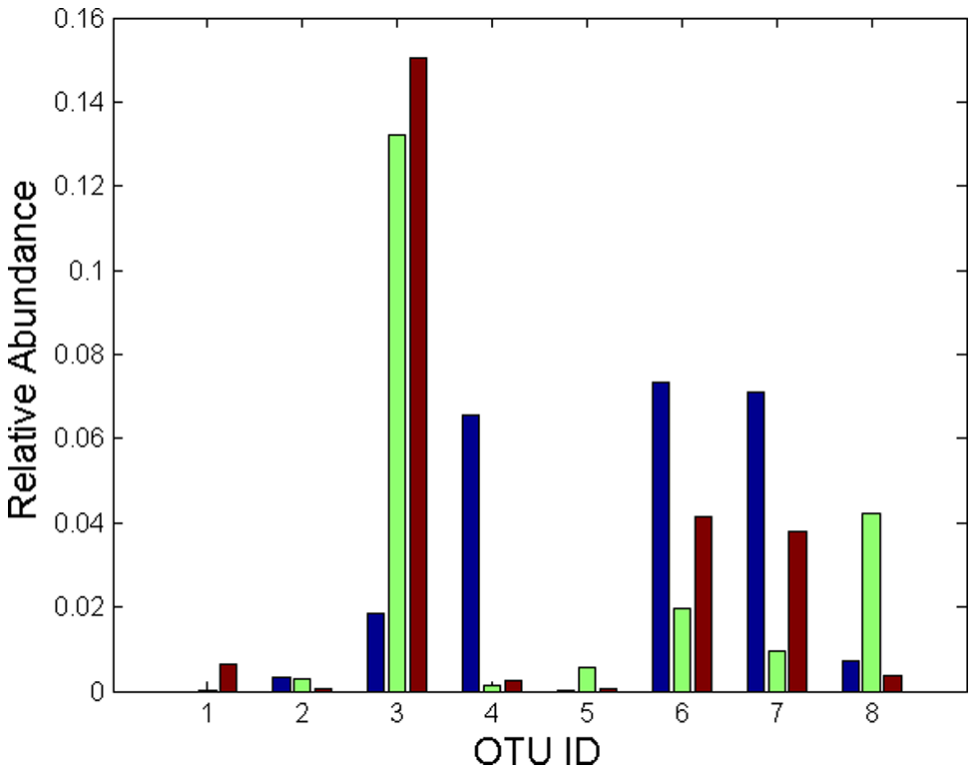
Relative abundances of the identified features for three healthy individuals: Left: Individual 1, Middle: 2, Right: 3.

The keyboard study dataset [39] was collected from three healthy individuals between 20 and 35 years of age. The keys of the three personal computer keyboards (25–30 keys per keyboard) and the skin on the ventral surface of the distal joint of each fingertip of the owner were swabbed for sample collection and microbial community analysis. There are total 104 samples with a sample size of 40, 33, 31 for each anonymous individual respectively. The main purpose of our study is to identify the OTUs that can distinguish the three experimental subjects correctly with our proposed method. We first normalized the data with proportion and arcsin transformation, and then evaluated the model performance with permutation and cross-validation. We partitioned the data into two parts, 2/3 of the data as training data and 1/3 of the data as test data. The free parameter 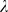 was determined by training data only. To prevent bias from a specific partition, we permutate the data 100 times. The identified OTUs are given in Table 3. The relative abundances of each identified OTU for three anonymous individuals are given in Figure 2.

**Table 3:** Identified OTUs for keyboard data.

ID	OTU Name
1	Bacteria;Firmicutes;Bacilli;Lactobacillales;Carnobacteriaceae (100)
2	Bacteria;Proteobacteria;Betaproteobacteria;Neisseriales;Neisseriaceae (88)
3	Bacteria;Actinobacteria;Actinobacteria;Actinomycetales;Propionibacteriaceae (100)
4	Bacteria;Actinobacteria;Actinobacteria;Actinomycetales;Corynebacteriaceae (100)
5	Bacteria;Actinobacteria;Actinobacteria;Actinomycetales;Micrococcaceae (100)
6	Bacteria;Firmicutes;Bacilli;Bacillales;Staphylococcaceae (100)
7	Bacteria;Firmicutes;Bacilli;Lactobacillales;Streptococcaceae (100)
8	Bacteria;Cyanobacteria;Chloroplast;Streptophyta (100)

With the free parameter of 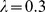, we identified 8 OTUs with predictive error of 0 and AUC of 100, which performs better than mlogit (test AUC 0.98) and is consistent with the best results reported by [40]. However, their approach requires 27 selected features (OTUs) to separate all samples of three anonymous individuals perfectly compared to ours with only 8 features. The 8 identified OTUs are from Actinobacteria, Cyanobacteria, Firmicutes, and Proteobacteria bacteria families respectively as shown in Table 3. In addition, both low abundance (Carnobacteriaceae, Neisseriaceae, and Micrococcaceae) and high abundance (such as Propionibacteriaceae) OTUS (genera) are highly differentiated in relative abundance across individuals as shown in Figure 2, demonstrating that hand-associated bacterial communities are highly diverse across individuals. Finally, the 8 identified OTUS (genera) can be used as potential biomarkers for forensic identification and medicine, especially given the fact that bacterial DNA is easier to recover than human DNA from the touched surfaces.

## Discussion

We have proposed LP based SVM approach with 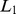/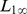 penalty for feature selection and binary/multiclass classification with applications to metagenomic count data. The proposed approach is easy to use since many large-scale free and commercial LP software are available in the literature. We demonstrated that the proposed approach performed well in simultaneously identifying features (OTUS) and predicting classes with limited experiments. Even though the proposed method was evaluated with 16 S metagenomic count data, it may be applicable to other types of genomic data (such as whole genome shotgun sequencing) in gene selection and classification with some simple modifications.
