# Correction: Class Prediction and Feature Selection with Linear Optimization for Metagenomic Count Data

**DOI:** 10.1371/journal.pone.0097958

**Published:** 2014-05-12

**Authors:** Zhenqiu Liu, Dechang Chen, Li Sheng, Amy Y. Liu

A reader recently pointed out certain similarities between the text in the article and that from some previous publications. Although our method in PLOS ONE was novel and citations to many of the relevant publications were included, the text should not have been used verbatim. The authors would like to thank the reader and apologize for the overlap in text.

The overlap in the text relates to the Introduction, Methods and Discussion sections of the article, where sentences from previous publications were reproduced, this relates to the following fragments in the text:

In the Methods section of the article there is some overlap in text with the first author Zhenqiu Liu’s previous publication, cited as reference 32:

Liu Z, Hsiao W, Cantarel B, Drbek E, Fraser-Liggett C (2011). Sparse distance based learning for simultaneous multiclass classification and feature selection of metagenomic data. Bioinformatics 27(23): 3242–3249.

The study reported in Bioinformatics and that published in PLOS ONE studied the same multiclass classification problem for metagenomic count data, and thus mathematical notations and the problem are defined in a similar way in the two articles. In addition, the description of the data normalization procedures in the PLOS ONE article is given in a way similar to that in the earlier publication which in Bioinformatics.

The description of the methods that overlap with that in the publication in Bioinformatics relates to the first paragraphs of the Methods section before the section titled and Penalized SVM Methods.

With regard to equations (6) and (7), our article cited the following article by Quattroni et al. as reference 35:

Quattoni A, Carreras X, Collins M, and Darrell T (2009). An Efficient Projection for L1,∞ Regularization, ICML 2009

However we acknowledge that it would have been more appropriate to cite this earlier work by the same group: Quattoni A, Collins M, and Darrell T (2008), Transfer Learning for Image Classification with Sparse Prototype Representations. In Proceedings of CVPR 2008.

Other instances of text overlap occur in the Introduction and in the Discussion sections of the article.

In the first paragraph of the Introduction, there are sentences that overlap with text in references 4 and 7, as well as Schloss et al. BMC Bioinformatics. 2008 Jan 23;9:34. doi: 10.1186/1471-2105-9-34, which was not cited in the article:

‘The majority of microbes reside in the gut, have a profound influence on human physiology and nutrition, and are crucial for human life. Metagenomics, the culture-independent isolation and characterization of DNA from uncultured microorganisms, has facilitated the analysis of the functional biodiversity harbored in the large reservoir of uncultured bacteria and archaea.’

‘Recent advances in genome sequencing technologies have made obtaining a complete metagenomic sequencing more tractable. Having on hand such a large number of microbial genomes has changed the nature of microbiology and of microbial evolution studies. By providing the ability to examine the relationship of genome structure and function across many different species, these data have also opened up the fields of comparative genomics and of systems biology. A main promise of metagenomics is that it will accelerate drug discovery and biotechnology by providing new genes with novel functions’

In the Results section, there are sentences that overlap with text from references 26 and 38 in the article:

‘ are simulated from the Gamma distribution with a mean (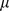) of 100 and variance (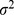) of 1000. We simulated 1000 features for each sample from NB distributions, which contained the first 5 relevant features having different distributions with distinguished s. We used two-fold cross validation to evaluate the method. First, we normalized the data with proportion and arcsin transformations, and then divided the data into training and test equal subsets. The training subset was used for model construction, while the test subset was used to evaluate performance. The model parameters are determined from only the training data with leave-one-out cross-validation.’

‘Bacteria thrive on and within the human body. One of the largest human-associated microbial habitats is the skin surface, which harbors large numbers of bacteria that can have important effects on health.’

Even though we cited the articles above, we apologize that the information was not rewritten more carefully.

The issue of existing text overlap has no bearing on the results and conclusions of the study.
